# Electron acceptors modulate methane oxidation and active methanotrophic communities in anoxic urban wetland sediments

**DOI:** 10.1128/aem.00386-25

**Published:** 2025-07-31

**Authors:** Ruiyu Yang, Chao Peng, Yongliang Mo, Sara Kleindienst, Shun Li, Jiajia Wang, Andreas Kappler, Zimeng Wang, Lu Lu

**Affiliations:** 1 College of Life Sciences, China West Normal University 56714, Nanchong, China; 2 Precise Synthesis and Function Development Key Laboratory of Sichuan Province, China West Normal University 56714, Nanchong, China; 3 College of Environmental Science and Engineering, China West Normal University 56714, Nanchong, China; 4 Department of Environmental Microbiology, Institute for Sanitary Engineering, Water Quality and Solid Waste Management (ISWA), University of Stuttgart 9149, Stuttgart, Germany; 5 State Key Laboratory of Microbial Technology, Shandong University 214177, Qingdao, Shandong, China; 6 School of Ecology, Shenzhen Campus of Sun Yat-Sen University 626367 , Shenzhen, Guangdong, China; 7 Geomicrobiology, Department of Geosciences, University of Tübingen 545687 , Tübingen, Germany; 8 Department of Environmental Science and Engineering, Fudan University 539573, Shanghai, China; University of Illinois Urbana-Champaign, Urbana, Illinois, USA

**Keywords:** anaerobic oxidation of methane, urban wetland, electron acceptors, DNA-stable isotope probing, metabolic mechanisms

## Abstract

Urban wetlands, although often overlooked, are hotspots for CH_4_ cycling. However, the understanding of anaerobic CH_4_ oxidation and microbial responses to different electron acceptors in urban wetlands remains limited. Here, we employed DNA-stable isotope probing (SIP) and metagenomic techniques to identify the core CH_4_-oxidizing microbial groups in the presence of NO_3_^-^, SO_4_^2-^, or ferrihydrite (Fe(III)) in anoxic urban-lake sediments. The addition of NO_3_^-^ to sediment microcosms promoted CH_4_ oxidation, whereas SO_4_^2-^ and Fe(III) had minimal or inhibitory effects on CH_4_ oxidation rates in anoxic microcosms. The SIP-identified methanotrophs in response to the addition of CH_4_ comprised the Marine Benthic Group D (MBG-D), “*Candidatus* Methylomirabilis Sh765B-TzT-35 and Z114MB74,” and the putatively aerobic methanotrophs *Methylobacter* and *Methylocystis*. The presence of NO_3_^-^ further enriched *Methylocaldum*, *Methylomonas*, ‘*Ca*. Methylomirabilis Sh765B-TzT-35 and Z114MB74’, *Methylococcaceae*, and *Methylospira*. In contrast, SO_4_^2-^ and Fe(III) amendments notably shifted the dominant methanotrophs through the significant labeling of *Methylocystis*. Furthermore, metagenomic analysis of ^13^C-labeled DNA showed enrichment of genes for oxidation of CH_4_ and its intermediates, as well as the reduction of NO_3_^-^, SO_4_^2-^, Fe(III), and fermentation. These results underscore the activity of NO_3_^-^-dependent CH_4_-oxidizing microorganisms and highlight their potential metabolic versatility in highly anthropogenically influenced urban wetlands.

**IMPORTANCE:**

Urban wetlands are critical ecosystems for CH_4_ cycling but are increasingly impacted by complex pollutants from urban development, such as nitrates, sulfates, and Fe(III) from industrial runoff, atmospheric deposition, and wastewater discharge. This study reveals how these pollutants act as electron acceptors, modulating microbial metabolic pathways and reshaping methanotrophic communities under anoxic conditions. By uncovering the microbial mechanisms driving CH₄ oxidation in urban wetland sediments, our findings provide a deeper understanding of how anthropogenic pollution alters biogeochemical cycles. These insights are crucial for developing targeted strategies to mitigate CH₄ emissions and improve greenhouse gas control in rapidly urbanizing regions.

## INTRODUCTION

Urban wetlands, such as artificial lakes or ponds, are widespread and represent persistent sources of CH_4_ emissions (1, 2). Their construction is increasing alongside urbanization to meet the growing demands for urban ecology and aesthetics (3, 4). The prevalence of the waterbodies and their substantial cumulative surface areas could even offset carbon uptake by the terrestrial carbon portion of the ecosystem (1, 5), a situation further exacerbated by high anthropogenic inputs of N, P, and labile organic matter (6, 7). Poor fluidity and insufficient dredging in urban wetlands rapidly consume dissolved oxygen at the sediment surface. This creates hypoxic or anoxic zones (8, 9), providing favorable conditions for active anaerobic oxidation of methane (AOM) that serve as a significant pathway for microbial CH_4_ removal (10). The unique characteristics of urban wetlands may lead to knowledge about CH_4_ oxidation patterns under anoxic conditions derived from natural water bodies being not directly applicable to urban waterbodies. However, most previous studies of AOM concentrated on natural waterbodies, such as lakes, reservoirs, and rivers (11, 12), with little understanding of AOM’s functional importance and active populations in urban wetlands.

Aerobic methanotrophs utilize CH_4_ monooxygenase (MMO) enzymes and O_2_ to convert CH_4_ into methanol, whereas anaerobic methane-oxidizing archaea and bacteria rely on electron acceptors like sulfate (SO_4_²⁻), manganese (Mn^4+^), iron (Fe(III)), nitrate (NO_3_⁻), or nitrite (NO_2_⁻) for CH_4_ oxidation (12–14). SO_4_²⁻-dependent AOM, involving “reverse” methanogenesis with sulfate-reducing *Deltaproteobacteria*, is crucial for mitigating CH_4_ emissions in high-sulfate marine environments (13, 15). Although SO_4_^2-^ concentrations are lower in freshwater than in marine environments, AOM coupled with SO_4_^2-^ reduction has been observed in low-salinity environments, even at typical freshwater SO_4_^2-^ levels (16, 17). SO_4_^2-^ concentrations in freshwater are continuously rising due to various human activities, including pharmaceutical production, paper manufacturing, mining, atmospheric deposition, and the application of SO_4_^2-^-containing fertilizer (18, 19). For instance, in the Canadian Prairies, SO_4_²⁻ levels have increased in 64% of the 14 lakes monitored in Saskatchewan over the past 30 years, which may affect CH_4_ cycling through complex mechanisms driven by elevated salinity (20). This trend underscores the importance of investigating AOM response to SO_4_^2-^ inputs in freshwater systems.

Unlike SO_4_^2-^, NO_3_^⁻^ and/or NO_2_^⁻^ is prevalent in freshwater environments, particularly in urban areas affected by city runoff or untreated sewage (21). NO_3_^⁻^/NO_2_^⁻^-dependent AOM has been shown to significantly reduce CH_4_ emissions from freshwater sediments (22, 23). In addition, Fe(III) reduction linked to AOM has been observed in iron-rich anoxic lake sediments, where methanotrophic bacteria utilize ferric oxides in partnership with Fe(III)-reducing bacteria (24). These reactions are thermodynamically more favorable than SO_4_^2-^-dependent AOM and yield more free energy under standard conditions (13). However, previous incubation experiments assessing the impacts of different electron acceptors on AOM in anoxic lake sediments have generated inconsistent results, indicating that our understanding of electron-acceptor-driven AOM remains incomplete (25–27). A recent study shows the presence of multi-electron acceptor-driven AOM in freshwater wetlands (28).

Regarding archaea, AOM is primarily conducted by three phylogenetic groups of anaerobic methane-oxidizing archaea (ANME) within the Euryarchaeota phylum: ANME-1 (with subgroups a and b), ANME-2 (with subgroups a, b, c), and ANME-3. Each phylogenetic ANME clade can be identified by distinct 16S rRNA and *mcrA* gene sequences (13, 29). ANME are anaerobic and commonly couple CH_4_ oxidation with SO_4_^2-^ reduction. The NO_3_^-^-reducing ANME strain *“Candidatus* Methanoperedens nitroreducens,” within the ANME-2d subgroup, has demonstrated enzymatic capabilities for reverse methanogenesis and can independently reduce NO_3_^-^ (30). The widespread distribution of ANME-2d in freshwater sediments suggests its significant role in CH_4_ oxidation within these environments (31). Methanotrophic bacteria affiliating with *“Candidatus* Methylomirabilis oxyfera“ (*Ca*. M. oxyfera), a member of the Methylomirabilota phylum (NC10), can aerobically oxidize CH_4_ under anoxic conditions by producing intracellular O_2_ from NO_2_^-^ via NO-dismutase (32). Many studies have observed the coupling of AOM to the reduction of Fe(III) and Mn(IV) based on biogeochemical evidence in lake sediments (16, 33).

Regarding aerobic methanotrophs, despite their dependence on O_2_ for CH_4_ oxidation, the activities of putatively aerobic methanotrophs have been detected in anoxic lake sediments (34). Methanotrophic bacteria (methanotrophic bacteria), especially those from the class Gammaproteobacteria, the order *Methylococcales*, appear to remain active in seemingly anoxic conditions (35). For example, labeling of DNA affiliated with *Methylobacter* (a gammaproteobacterial methanotrophic bacteria) and Fe(III)-reducing bacteria in ^13^CH_4_-based SIP experiments with sub-Arctic lake sediments indicates that Fe(III)-reducers assimilate organic compounds released by methanotrophic bacteria under anoxic conditions (36). A recent study suggests that methanotrophic bacteria can utilize fermentation-based methanotrophy and denitrification under anoxic conditions, providing an explanation for their widespread occurrence in anoxic environments (37). However, it remains unclear whether methanotrophic bacteria contribute to CH_4_ oxidation in anoxic urban lake sediments.

We hypothesize that urban lakes receive various pollutants that can alter the redox conditions and nutrient profiles, potentially affecting the process of CH_4_ oxidation under anoxic conditions. Thus, this study aimed to (i) identify the active methanotrophs and their potential metabolic pathways and (ii) examine their response to the addition of different electron acceptors (NO_3_^-^, SO_4_^2-^, Fe(III)) in sediments sampled from an urban lake with relatively high CH_4_ production (see Fig. S1 at https://doi.org/10.6084/m9.figshare.29396813). DNA-SIP, coupled with metagenomics and amplicon sequencing, was used to analyze metabolically active methanotrophs in ^13^C-labeled DNA derived from anoxic microcosms. Geochemical parameters, including the production of ^13^CO_2_ from ^13^CH_4_ and the concentration of electron acceptors, were also monitored to correlate with molecular data in lake sediments.

## MATERIALS AND METHODS

### Site description and sediment collection

Sediment samples were collected from Yingxi Lake, an urban lake in Nanchong City, China (106°03′46″E, 30°49′15″N). Nanchong, a rapidly urbanizing city with a population exceeding 7.6 million, has experienced heightened demand for green spaces, leading to the construction of many artificial lakes. Despite being constructed 15 years ago, these lakes suffer from anthropogenic pollution, primarily due to improper waste disposal and poor runoff management.

Sediment samples were collected from the top 0–15 cm at the bottom of Yingxi Lake (water depth: ~4 m) using a grab-type sampler. Three random subsamples were taken, sealed in sterile ziplock bags, and transported to the laboratory in a cooler at 4°C. Additionally, lake water samples were also collected. The water samples were filtered with a filter membrane with a pore size of 0.22 µm and deoxidized with N_2_ gas (99%) for 30 min. The sediment samples and deoxidized water samples were stored at 4°C for the subsequent microcosm experiment within 1 day.

### DNA-SIP microcosm setup

The microcosm setups were performed in an anoxic glovebox (99.999% N_2_, O_2_ < 0.1 ppm, Mbraun Unilab Pro, Germany). Each 120 mL serum bottle was amended with ~14 g of sediment (equivalent to 10.0 g dry weight soil (*d.w.s*)) and 20 mL of deoxygenated lake water, then sealed with butyl rubber stoppers (Chemglass Life Sciences, Vineland, NJ). Pre-incubation was conducted to exhaust any O_2_ potentially carried over from the sediments. The microcosms were preincubated without any amendments. To monitor O_2_ concentration changes during the pre-incubation, two separate redox indicator treatments were set up with the amendment of resazurin (1 mg/L) or resazurin and Na_2_S_2_O_3_ (1 mM). Shifts from pink to colorless in the lake water indicated an anoxic or reduced environment within the bottles (38). The pre-incubation lasted for 5 days until the overlying river water in the microcosms turned colorless (see Fig. S2 at https://doi.org/10.6084/m9.figshare.29396813). The actual incubations were then initiated by adding either Candoornd electron acceptors to new microcosms set up in the same way but without resazurin or Na_2_S_2_O_3_.

Nine treatments were prepared: (i) sediment without additions (CK), (ii) ^13^CH_4_-labeled microcosms supplied as '^13^CH_4_', (iii) ^12^CH_4_-control microcosms supplied as '^12^CH_4_', (iv) ^13^CH_4_-labeled amended with 5 mM NaNO_3_, supplied as '^13^CH_4^-^_+NO_3_^-^', (v) ^12^CH_4_-control amended with 5 mM NaNO_3_, supplied as '^12^CH_4^-^_+NO_3_^-^', (vi) ^13^CH_4_-labeled amended with 5 mM Na_2_SO_4_, supplied as '^13^CH_4_+SO_4_^2-^', (vii) ^12^CH_4_-control amended with 5 mM NaSO_4_^2-^, supplied as '^12^CH_4_+ SO_4_^2-^', (viii) ^13^CH_4_-control amended with 10 mM ferrihydrite, supplied as '^13^CH_4_ +Fe(III)', and (ix) ^12^CH_4_-control amended with 10 mM ferrihydrite, supplied as '^12^CH_4_ +Fe(III). ^13^CH_4_ (99 atom % ^13^C, Sigma-Aldrich, USA) or CH_4_ (99.99% purity, as unlabeled control) was injected to obtain a headspace concentration of 5% CH_4_ (vol/vol). All treatments were performed in triplicates. The bottles were incubated at 25°C in darkness for 110 days. About 10 g of sediment sample per bottle was harvested and immediately flash-frozen in liquid nitrogen and stored at −20°C for molecular analysis. The remaining sediment was homogenized and centrifuged at 5,000 rpm for 10 min. The supernatant was filtered through a 0.45 µm membrane to determine concentrations of NO_3_^-^, SO_4_^2-^, and Fe(II). Concentrations of NO_3_^-^ and SO_4_^2-^ were measured using ion chromatography (ICS-2100, Dionex, USA). Total Fe(II) in the samples was extracted with 1 M HCl overnight, mixed with ferrozine, and then quantified at the wavelength of 562  nm on a spectrophotometer (39).

### Quantification of CH_4_ and ^13^C-CO_2_ in the headspace

To monitor changes in CH_4_ concentration, headspace gas samples (200 µL) were withdrawn from the microcosms after 0, 8, 28, 60, and 110 days using a syringe flushed with N_2_ (99.999% purity) three times to remove trace amounts of O_2_. The concentration of CH_4_ was measured by gas chromatography with a thermal conductivity detector (Shimadzu GC-14B, Japan). The collected gas vials were analyzed for δ^13^CO_2_ value with a continuous flow isotopic ratio mass spectrometer (CF-IRMS; Delta V Advantage, Thermo Fisher Scientific, Bremen, Germany) coupled with a gas handling device (Gas-Bench II; Thermo Fisher Scientific, Bremen, Germany). The ^13^CO_2_ concentrations in the gas samples were calculated according to the formula δ^13^C(‰) = (R_sample_/R_standard_^−1^)*1,000, R = ^13^C/^12^C, the δ^13^C value is calibrated based on the VPDB (Vienna Pee Dee Belemnite) standard, and the R_standard_ value was 0.0112372.

### DNA extraction and SIP gradient fractionation

Total DNA was extracted from each ~0.5 g slurry sample using the FastDNA SPIN Kit for Soil (MP Biomedicals, Cleveland, OH, USA) according to the manufacturer’s instructions. The concentration and quality of extracted DNA were evaluated with a UV-VIS spectrophotometer (NanoDrop Technologies, Wilmington, DE, USA), and DNA was then stored at −20°C for subsequent analysis.

Density gradient ultracentrifugation was performed for each treatment to separate the ^13^C-labeled DNA from the total DNA in SIP microcosms as described previously (40). Briefly, 3 µg of DNA was mixed with the CsCl stock solution. The DNA mixture buoyant density was adjusted to 1.725 g mL^−1^ using the gradient buffer (0.1 M Tris-HCl, 0.1 M KCl, 1 mM EDTA, pH 8.0). The solution was transferred to a polyallomer bell-top ultracentrifuge tube (5.1 mL, Beckman Coulter, Pasadena, CA, USA) and heat-sealed. The DNA mixture was spun on an NVT 65.2 rotor (Beckman Coulter, USA) at 177,000 × g (45,000 rpm) for 44 h at 20°C. The centrifuged gradient was fractionated from bottom to top by displacing the gradient medium with sterile water using a syringe pump NE-1000 (New Era Pump System, USA). A total of 14 DNA gradient fractions of equal volumes (~ 340 µL) were generated, and 70 µL liquid of each fraction was used for refractive index measurement using an AR200 digital hand-held refractometer (Reichert, Buffalo, NY, USA). The DNA was separated from the CsCl solution by precipitation using 30% polyethylene glycol (PEG) 6000 and purified with 70% ethanol. The fractionated DNA was dissolved in 30 µL TE buffer and stored at −20°C.

### Real-time quantitative PCR of *mcrA* and *pmoA* genes

Real-time quantitative PCR (qPCR) assays for the *pmoA* gene were conducted in triplicate to quantify the abundance of genes targeting anaerobic CH_4_-cycling archaea and aerobic CH_4_-oxidizing bacteria and verify ^13^C assimilation into the genomic DNA of involved microorganisms by analyzing distribution patterns throughout the SIP gradients. The qPCR analyses were performed on a CFX9Z Optical Read-time Detection System (Bio-Rad Laboratories Inc., Hercules, CA, USA). The qPCR standards were generated using plasmid DNA from one representative clone containing *mcrA* or *pmoA*. A standard template dilution series with seven 10-fold gradients was used. The qPCR conditions and primer are described in Table S1 at https://doi.org/10.6084/m9.figshare.29396813. The amplification efficiencies of 98%–103% were obtained with R^2^ values of 0.993–0.999.

### Illumina MiSeq sequencing of the 16S rRNA gene

The microbial community compositions were analyzed using Illumina MiSeq sequencing of the V4 region of the 16S rRNA gene. The 16S rRNA gene fragment was amplified using primers ArBa515f and Arch806r (41, 42). Library preparation (Nextera, Illumina) and 250 bp paired-end sequencing with Miseq (Illumina, San Diego, CA) using *v*2 chemistry were performed. Quantitative Insights Into Microbial Ecology (QIIME2) was used to process and analyze the adapter and primer-free sequences (43). Short, low-quality, ambiguous barcodes (one or more mismatching bases) nucleotide reads were removed using Cutadapt and DADA2 (44, 45). Amplicon sequence variants (ASVs) were taxonomically assigned using QIIME2′s “q2-feature-classifier” trained on the Silva database (nr_v132). FAPROTAX prediction was analyzed to infer the function profiles of the microbial communities (46).

### Metagenomics sequencing analysis

The identified heavy ^13^C-labeled DNA fractions in ^13^CH_4_-amended treatments (supplied as ^13^C-DNA) and the corresponding fractions from the ^12^CH_4_-amended treatments (supplied as ^12^C-DNA) were collected for metagenomic analysis. Due to the low DNA amount and quality in SIP fractions, the identified heavy fractions of the triplicate sample in each treatment were pooled into one sample for metagenomics sequencing. Genomic DNA was fragmented using sonication and subjected to shotgun metagenomic analysis by an Illumina NovaSeq 6000 platform. Paired-end fragment libraries with an insert size of 400  bp were constructed using NEXTFLEX Rapid DNA-Seq (Bioo Scientific, Austin, TX, USA). Raw sequencing reads were filtered to obtain high-quality data for subsequent analysis using Fastp (47) (https://github.com/OpenGene/fastp, version 0.20.0). In total, 95% of reads were filtered with SOAP denovo2 (48). The gene catalogs were aligned against the KEGG database (89.1) and CARD database (4.0) using DIAMOND v0.9.19 (http://www.diamondsearch.org/index.php, version 0.8.35) with an *e*-value cutoff of 1e^−5^. The relative abundance of functional genes was calculated using the TPM (transcripts per million) normalization method, which normalizes read counts by gene length and total mapped reads. TPM values were obtained by normalizing the number of reads mapped to each gene by its length (in kilobases) and the total number of mapped reads in the sample.

### Data analysis

All data were analyzed using Microsoft Excel 2019 and SPSS Statistics 25.0 (IBM Inc., Chicago, IL, USA). Origin 2018 (Origin Lab Inc., USA) and the R package “ggplot2”(R 4.2.0) were used for data visualization. Phylogenetic analysis was carried out in Mega 11 software. LEfSe combines the standard tests for statistical significance (Kruskal–Wallis test and pairwise Wilcoxon test) with linear discriminant analysis to identify the key CH_4_ oxidizers in different treatments by comparing the microbial communities of SIP-fractioned DNA in ^12^C-CH_4_ and ^13^C-CH_4_-amended treatments.

## RESULTS

### CH_4_ consumption and ^13^CO_2_ production in experiments amended with different electron acceptors

**Fig 1:**
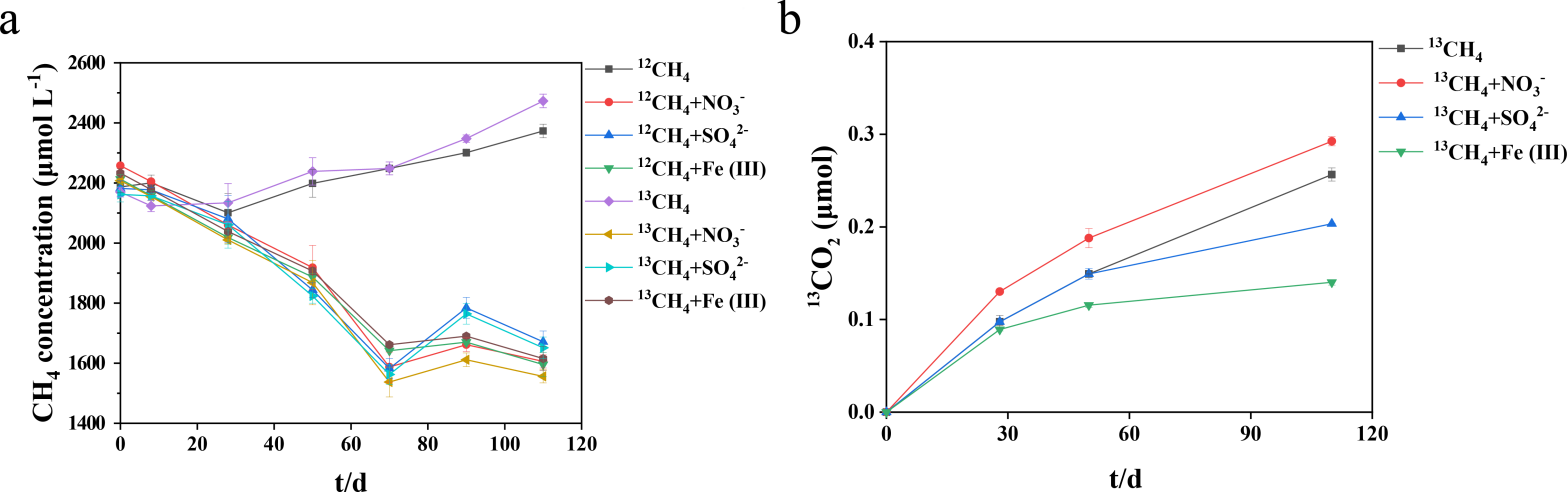
Effects of electron acceptor additions on CH_4_ emission and CH_4_ oxidation during anoxic incubations. (**a**) Changes in CH_4_ concentrations in the headspace of the microcosms under different treatments after incubation for 0, 8, 28, 50, 70, 90, and 110 days. The treatments include microcosms amended with ^12^CH_4_, ^12^CH_4_+ NO_3_^-^, ^12^CH_4_+ SO_4_^2-^, ^12^CH_4_ + Fe (III), ^13^CH_4_, ^13^CH_4_+ NO_3_^-^, ^13^CH_4_+ SO_4_^2-^, and ^13^CH_4_ + Fe (III). (**b**) The concentration of ^13^CO_2_ derived from ^13^CH_4_ in the headspace of microcosms amended with ^13^CH_4_, ^13^CH_4_+ NO_3_^-^, ^13^CH_4_+ SO_4_^2-^, and ^13^CH_4_ + Fe (III) after incubation for 0, 28, 50, and 110 days. Error bars represent the standard deviation from three biological replicates.

The sediment used in this study exhibited high methanogenic activity, with methane production rates reaching up to 2.0 µg CH₄ g^−1^ d^−1^ under anaerobic incubation between days 28 and 50, but this activity was significantly suppressed by the addition of different electron acceptors (see Fig. S1 at https://doi.org/10.6084/m9.figshare.29396813). In CH_4_-amended microcosms, the CH_4_ concentrations in the headspace of the bottles also decreased to varying degrees with the addition of different electron acceptors (*P* < 0.01) (Fig. 1). The AOM rates were estimated using the slope from the linear regression (see Fig. S3 at https://doi.org/10.6084/m9.figshare.29396813), with observed rates ranging from 0.15 to 0.38 µg C g^−1^ d^−1^.

The activity of AOM was further evaluated through the increase in the amount of ^13^CO_2_ derived from ^13^CH_4_ (Fig. 1b). Significant ^13^CO_2_ production was detected across all ^13^CH_4_-amended treatments, with the highest ^13^CO_2_ production occurring in the ^13^CH _4_+ NO_3_^-^ treatment, followed by ^13^CH_4_, ^13^CH _4_+ SO_4_^2-^, and ^13^CH_4_ + Fe(III) treatments. Notably, the ^13^CH _4_+ NO_3_^-^ treatment exhibited higher ^13^CO_2_ production, compared with the ^13^CH_4_ treatment, with an increase of 26.0% and 14.0% at days 50 and 110, respectively. Conversely, the addition of SO_4_^2-^ and Fe(III) did not stimulate ^13^CO_2_ production. In parallel, the reduction of NO_3_^-^ and SO_4_^2-^ and the production of Fe(II) were observed in CH_4_ microcosms with NO_3_^-^, SO_4_^2-^ and Fe(III) additions (see Fig. S4 at https://doi.org/10.6084/m9.figshare.29396813). It was noted that although a clear decrease in CH_4_ concentration was observed after the addition of electron acceptors, and ^13^CO_2_ production indicated the occurrence of methane oxidation, the quantity of ^13^CO_2_ was relatively small compared to the decrease of CH_4_.

### Changes in abundances of mcrA and pmoA genes in different microcosm experiments

**Fig 2:**
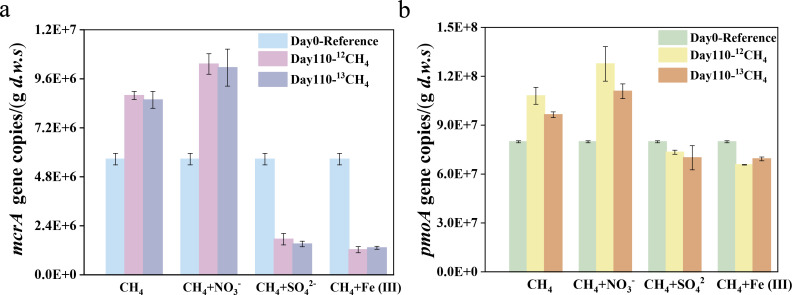
Changes in copy numbers of the archaeal *mcrA* gene (**a**) and bacterial *pmoA* gene (**b**) in anoxic microcosms amended with CH_4_, CH _4_+ NO_3_^-^, CH _4_+ SO_4_^2-^, and CH_4_ + Fe (III) during the incubation period of 110 days. The data displayed in this figure show no significant differences between treatments amended with ^13^CH_4_ and ^12^CH_4_. Error bars represent the standard deviation from three biological replicates. The designations are the same as those in Fig. 1.

In the ^12^CH_4_/^13^CH_4_ microcosms without the addition of any electron acceptors, the copy numbers of *mcrA* and *pmoA* genes increased significantly from 5.7 × 10^6^ and 8.0 × 10^7^ at day 0, to 8.7 × 10^6^ and 1.02 × 10^8^ copies· (g *d.w.s*)^−1^ after incubation for 110 days (*P* < 0.05), representing approximately 1.5-fold and 1.3-fold increases, respectively (Fig. 2). The addition of CH_4_+NO_3_^-^ resulted in significantly higher abundances of *mcrA* and *pmoA* genes at day 110, with increases of 19.0% and 24.9%, respectively, compared with the CH_4_ treatment (*P* = 0.001). In contrast, *mcrA* gene copies decreased by 69.2% and 78.3% at day 110 in CH_4_+ SO_4_^2-^ and CH_4_ + Fe(III) treatments, respectively, along with a slight decrease in the *pmoA* gene copy number. The decrease in *mcrA* gene abundance, coupled with the reduced CH_4_ production, suggests that the addition of SO_4_^2-^ and Fe(III) likely inhibited the activity of methanogenic archaea.

### Changes in the microbial diversity and potential methanotrophic communities

**Fig 3:**
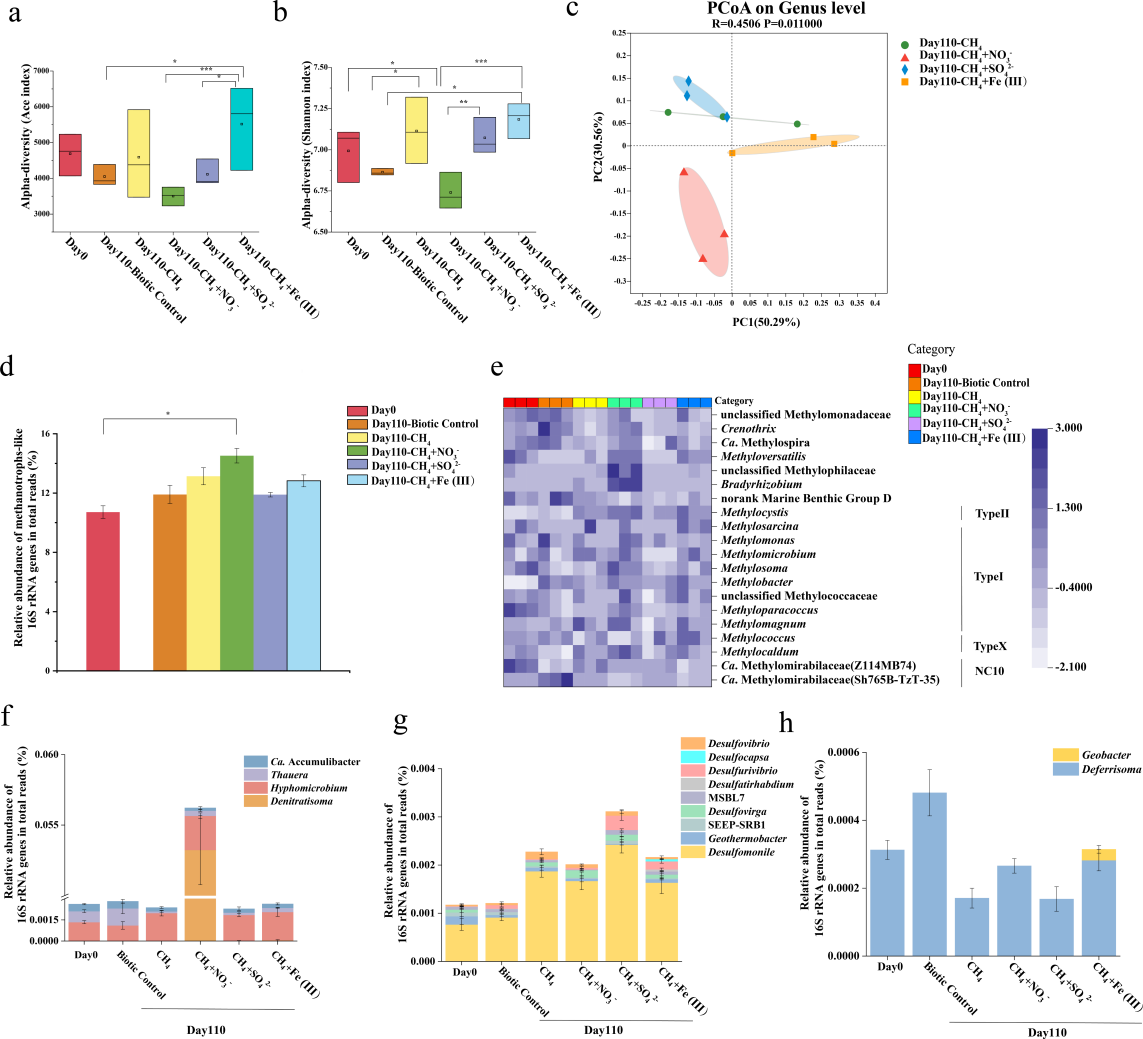
Changes in overall microbial communities and key functional groups in anoxic microcosms over the incubation period. (**a and b**) Changes in alpha diversity indexes Ace and Shannon in Biotic Control, CH_4_, CH_4_+ NO_3_^-^, CH_4_+ SO_4_^2-^, and CH_4_ + Fe (III) treatments after incubation for 0 and 110 days. The horizontal black lines and stars within the boxes represent the median and the mean, respectively. Statistically different treatments (pair-wise ANOVA) are connected by bracket, and the level of significance is shown with *(*P* < 0.05), **(*P* < 0.01), or ***(*P* < 0.001). (**c**) Principal coordinate analysis plot of the microbial community structure of different treatments based on Bray-Curtis distances. The percentage of variance explained by each principal coordinate axis is shown in parentheses. (**d**) The relative abundance of methanotrophs-like 16S rRNA gene reads to the total DNA reads in the Biotic Control, CH_4_, CH_4_+ NO_3_^-^, CH_4_+ SO_4_^2-^ and CH_4_ + Fe (III) treatments after incubation for 0 and 110 days. (**e**) The heatmaps are based on the relative abundance of potential methane-oxidizing microorganisms in different treatments. (**f**) Relative abundances of the main denitrifying bacteria, (**g**) sulfate-reducing bacteria, (**h**) and iron(III)-reducing bacteria in different treatments. The designations are the same as those in Fig. 1.

After incubation for 110 days, diversity indices (Ace and Shannon index) were lowest in the CH_4_+ NO_3_^-^ treatment, and the microbial composition was significantly different from that of all other treatments (*P* < 0.05) (Fig. 3a and b). Principle coordinates analysis (PCoA) plot based on Bray-Curtis dissimilarities revealed distinct clustering of treatments (*P* < 0.05) (Fig. 3c). PERMANOVA analysis further confirmed that treatments were significant contributors to beta-diversity variance, explaining up to 54.6% of the variability (R^2^ = 0.546, *P* = 0.018) (see Table S2 at https://doi.org/10.6084/m9.figshare.29396813).

The relative abundance of methanotroph-like 16S rRNA genes was highest in the CH_4_+ NO_3_^-^ treatment, showing a 36.0% increase compared with Day 0 (Fig. 3d). This pattern aligned with the CH_4_+ NO_3_^-^ treatment exhibiting the highest ^13^CO_2_ production. These concurrent increases suggest enhanced methanotrophic activity in the CH_4_+ NO_3_^-^ treatment. In the CH_4_ and CH_4_ + Fe(III) treatments, it increased from 10.7% to 13.1% and 12.8%, respectively, after incubation for 110 days. Besides, the changes in the relative abundance of methanotroph-like 16S rRNA genes were significantly positively correlated with ^13^CO_2_ production across all treatments (see Fig. S5 at https://doi.org/10.6084/m9.figshare.29396813).

The compositions of methanotrophic communities and predicted metabolic pathways varied among treatments (Fig. 3e; see Fig. S6 at https://doi.org/10.6084/m9.figshare.29396813). In the CH_4_ treatment, the relative abundance of *Methylobacter* and *Methylocystis* increased by 2.4-fold and 2.7-fold, respectively. Enrichment of *Bradyrhizobium* and *Methylocystis* was observed in the CH_4_+NO_3_^-^ treatment.

### Microbial shifts in microorganisms related to denitrification, sulfate reduction, and iron(III) reduction

The enrichment of the denitrifying bacterium *Denitratisoma* was only detected in the CH_4_+NO_3_^-^ treatment, where its abundance reached 5.3% at day 110 (Fig. 3f). The highest relative abundance of sulfate-reducing bacteria *Desulfomonile* and *Desulfurivibrio* was observed in the CH_4_+ SO_4_^2-^ treatment (Fig. 3g). *Geobacter* was exclusively enriched in the CH_4_ +Fe(III) treatment (Fig. 3h).

### ^13^C-labeled active methanotrophic microorganisms in different treatments

**Fig 4:**
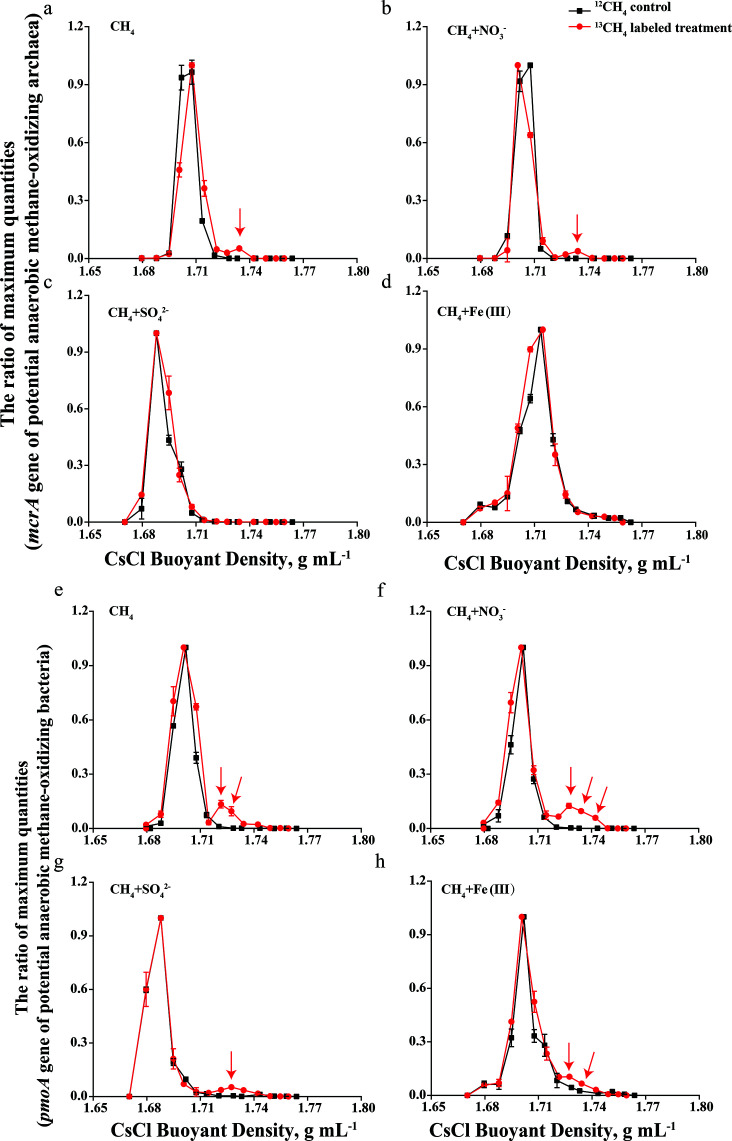
Relative abundance distribution of the archaeal *mcrA* genes (**a–d**) and the bacterial *pmoA* genes (**e–h**) in the fractionated DNA across the CsCl density range of the SIP gradients from sediments microcosms amended with CH_4_ (**a and e**), CH_4_+ NO_3_^-^ (**b and f**), CH_4_+ SO_4_^2-^ (**c and g**), and CH_4_ + Fe (III) (**d and h**) over the incubation course of 110 days. The normalized data are the ratios of the gene copy number in each DNA gradient to the maximum quantities from each treatment. The standard error of the triplicate samples is shown, with some error bars smaller than the symbol size. Filled circles in red represent ^13^CH_4_ labeled treatment, and black filled circles represent ^12^CH_4_ control.

qPCR analysis revealed notable labeling of *mcrA* and *pmoA* genes in CH_4_ and CH _4_+ NO_3_^-^ treatments (Fig. 4). In all microcosms, the copy number of *mcrA* and *pmoA* genes peaked in the “light” fractions with the buoyant density of 1.68–1.70 g mL^−1^, typical for the unlabeled DNA. However, in ^13^CH_4_ and ^13^CH_4_+ NO_3_^-^ treatments, a second peak in the ^13^CO_2_-labeled microcosms was observed in the “heavy” DNA fractions with buoyant densities of 1.72–1.74 g mL^−1^ (fractions 5–7, ranging from dense to light fractions) ( Fig. 4a, b, e, and f), suggesting the labeling of genomes involved in CH_4_ oxidation over the 110-day incubation. In the CH_4_+ SO_4_^2-^ and CH_4_ + Fe(III) microcosms, the peak in the “heavy” DNA fractions was only obtained for *pmoA* genes, but not obvious for *mcrA* gene.

**Fig 5:**
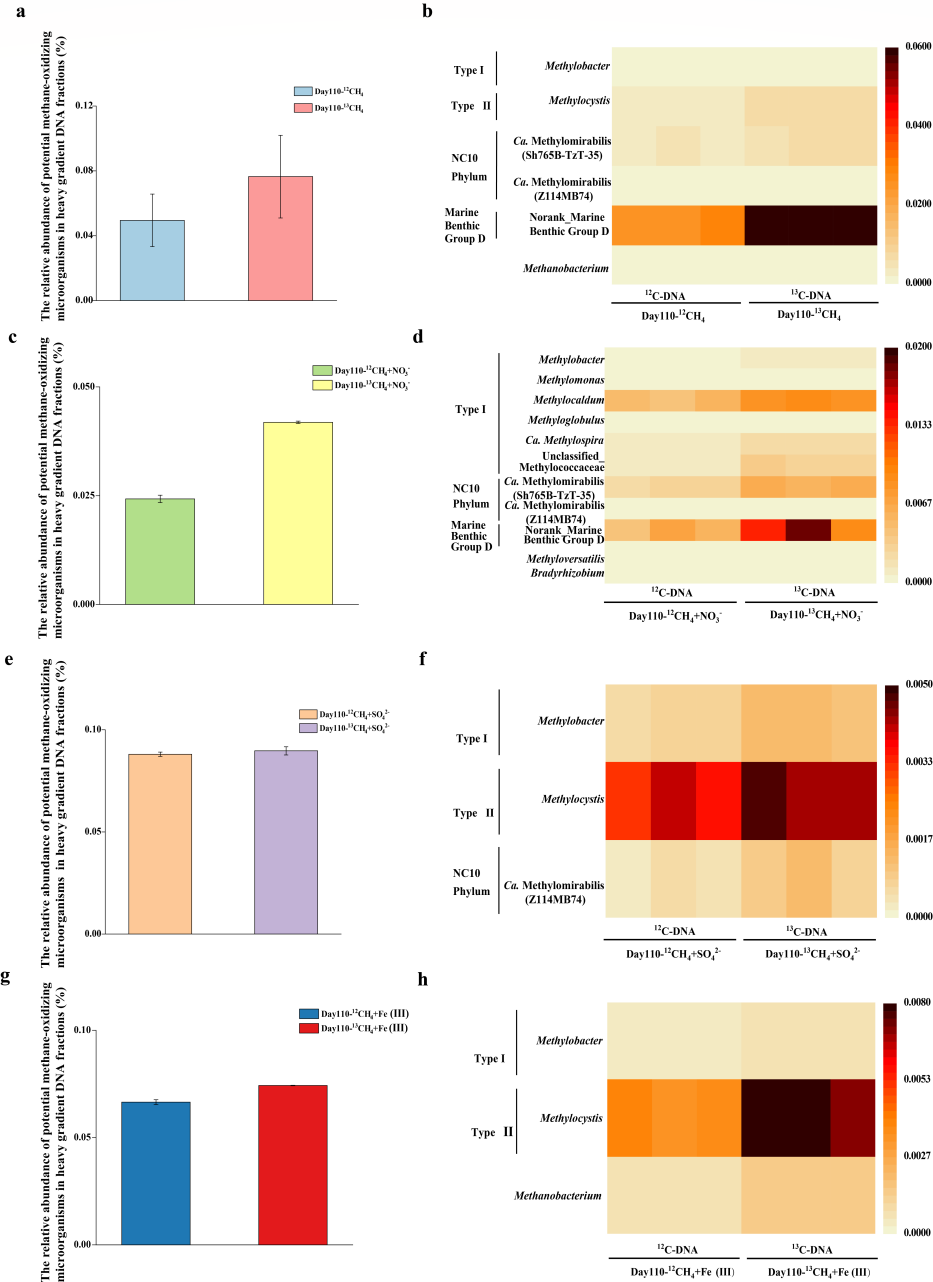
Relative abundance of methanotroph-like 16S rRNA gene reads to the total DNA reads in the fractionated ^13^C-DNA and ^12^C-DNA in microcosms amended with ^12^CH_4_ /^13^CH_4_ (**a**), ^12^CH_4_ /^13^CH_4_ + NO_3_^-^ (**c**), ^12^CH_4_ /^13^CH_4_ + SO_4_^2-^ (**e**), and ^12^CH_4_ /^13^CH_4_ + Fe (III) (**g**) treatments after incubation for 110 days. Error bars represent the standard deviation from three biological replicates. The heatmap patternsshowg significant enriched methanotrophic microorganisms in ^13^C-DNA compared with ^12^C-DNA in microcosms amended with ^12^CH_4_ /^13^CH_4_ (**b**), ^12^CH_4_ /^13^CH_4_ + NO_3_^-^ (**d**), ^12^CH_4_ /^13^CH_4_ + SO_4_^2-^ (**f**), and ^12^CH_4_ /^13^CH_4_ + Fe (III) (**h**) treatments after incubation for 110 days. The numbers in the scales show the relative abundance of methane-oxidizing microorganisms.

Miseq sequencing analysis of the total microbial community was performed on "heavy" ^13^C-DNA gradient fraction 5–7 from ^13^CH_4_-labeled microcosms and the control ^12^C-DNA from ^12^CH_4_ microcosms at day 110 (Fig. 5). Enrichment of methanotrophs-like 16S rRNA gene sequences was observed in all ^13^C-DNA samples. In CH_4_ treatments, 7.7% of the total 16S rRNA genes in the ^13^C-DNA were assigned to methanotrophic communities, whereas only 5.0% were found in the ^12^C-DNA. In CH_4_+ NO_3_^-^ microcosms, the proportion of methanotrophs-like 16S rRNA genes increased from 2.4% in the ^12^C-DNA to 4.2% in the ^13^C-DNA. In contrast, CH _4_+ SO_4_^2-^ and CH_4_ + Fe(III) additions led to only a slight increase in the proportion of methanotrophs-like 16S rRNA gene in the ^13^C-DNA, respectively, after incubation for 110 days.

### Phylogenetic analysis of active methanotrophs in sediments

**Fig 6:**
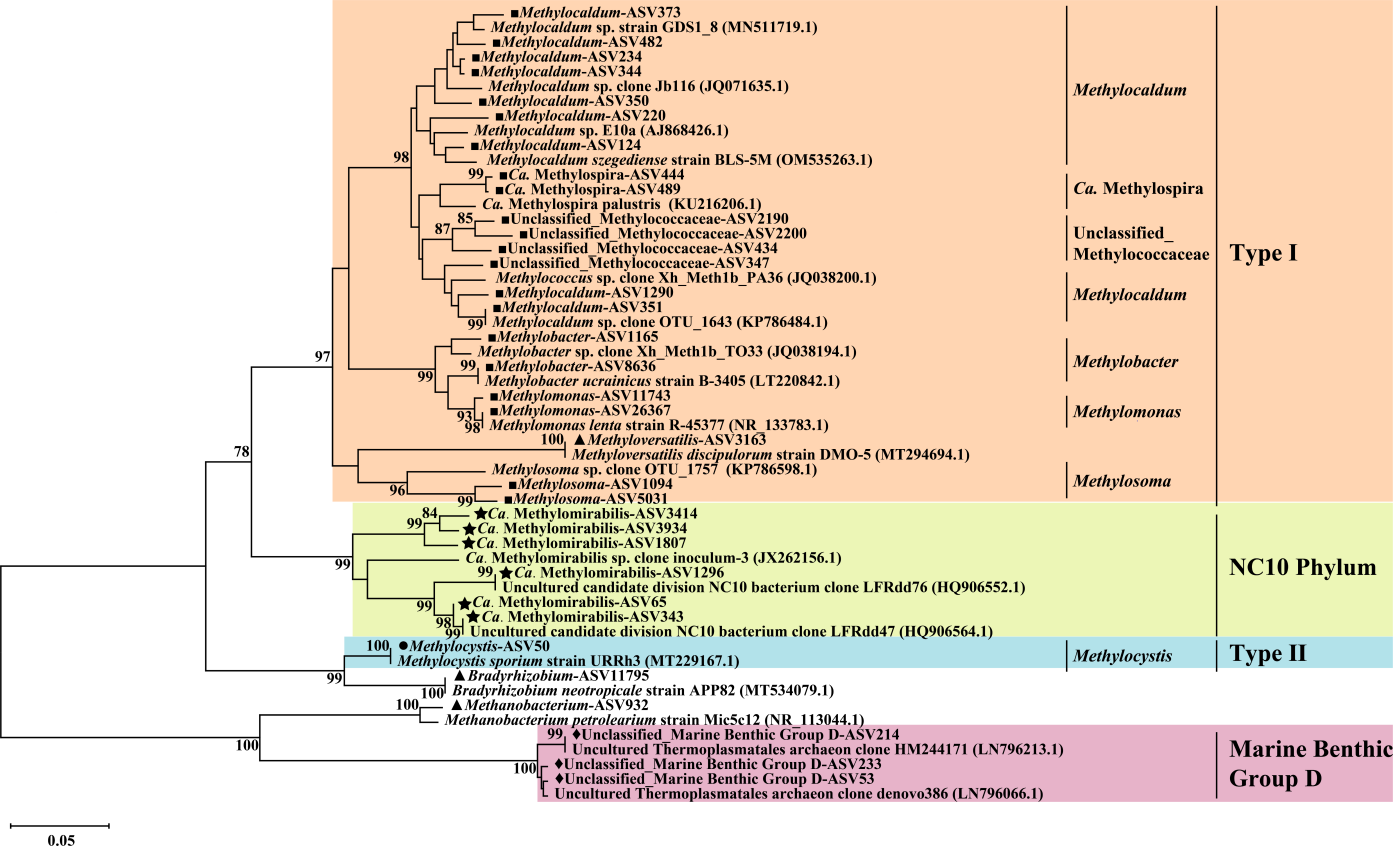
Phylogenetic tree of representative 16S rRNA gene sequences directly amplified from the heavy DNA. The scale bar represents 0.05 substitutions per nucleotide position. Bootstrap values > 70 are shown. Different color blocks characterize different classes of methane-oxidizing microorganisms.

Phylogenetic analysis of 16S rRNA gene sequences showed that the dominant active methanotrophic communities varied across treatments (Fig. 5 and 6; also see Fig. S7 at https://doi.org/10.6084/m9.figshare.29396813). Notably, the enrichments of Marine Benthic Group D (MBG-D)*, “Candidatus* Methylomirabilis Sh765B-TzT-35” (*Ca*. Methylomirabilis Sh765B-TzT-35), *“Ca*. Methylomirabilis Z114MB74,” *Methylobacter*, and *Methylosinus* were detected in the ^13^C-DNA of the ^13^CH_4_ microcosms. These enrichments showed enrichment factors of 2.4, 1.6, 2.6, 1.8, and 2.0, respectively, compared with the ^12^CH_4_ microcosms. MBG-D 16S rRNA genes accounted for up to 6.3% of the total sequence reads in the ^13^C-DNA of the ^13^CH_4_ treatment. Notably, MBG-D predominated in both the ^13^CH_4_ and ^13^CH_4_+ NO_3_^-^ treatments, representing 79.09% and 42.91% of methanotrophic-like 16S rRNA gene reads, respectively. Network analysis of microbial communities across all treatments further identified MBG-D as a key taxon exhibiting high connectivity with other microorganisms (see Fig. S8 and Table S3 at https://doi.org/10.6084/m9.figshare.29396813).

The addition of CH_4_+NO_3_^-^ further facilitated a more diverse set of active methanotrophic communities, including MBG-D, Type I methane-oxidizing bacteria, including *Methylomonas*, *Methylocaldum*, *Methylobacter*, *Methylosoma*, *Methylospira*, *unclassified Methylococcaceae*, as well as NC10 bacteria “*Ca*. Methylomirabilis Sh765B-TzT-35” and “*Ca*. Methylomirabilis Z114MB74.” Additionally, novel methanotrophs *Methyloversatilis* and *Bradyrhizobium* were also labeled in ^13^C-DNA of the ^13^CH_4_+ NO_3_^-^ treatment.

In CH_4_+ SO_4_^2-^ and ^13^CH_4_ + Fe(III) treatments, *Methylobacter* and *Methylocystis* were enriched in ^13^C-DNA samples. ‘Ca. *Methylomirabilis* Z114MB74’ was labeled in the ^13^C-DNA of the CH_4_+ SO_4_^2-^ treatment. Furthermore, the methanogen *Methanobacterium* was enriched in the ^13^C-DNA of both ^13^CH_4_ and ^13^CH_4_ + Fe(III) treatments.

### Metagenomic analysis of ^13^C-DNA and ^12^C-DNA

**Fig 7:**
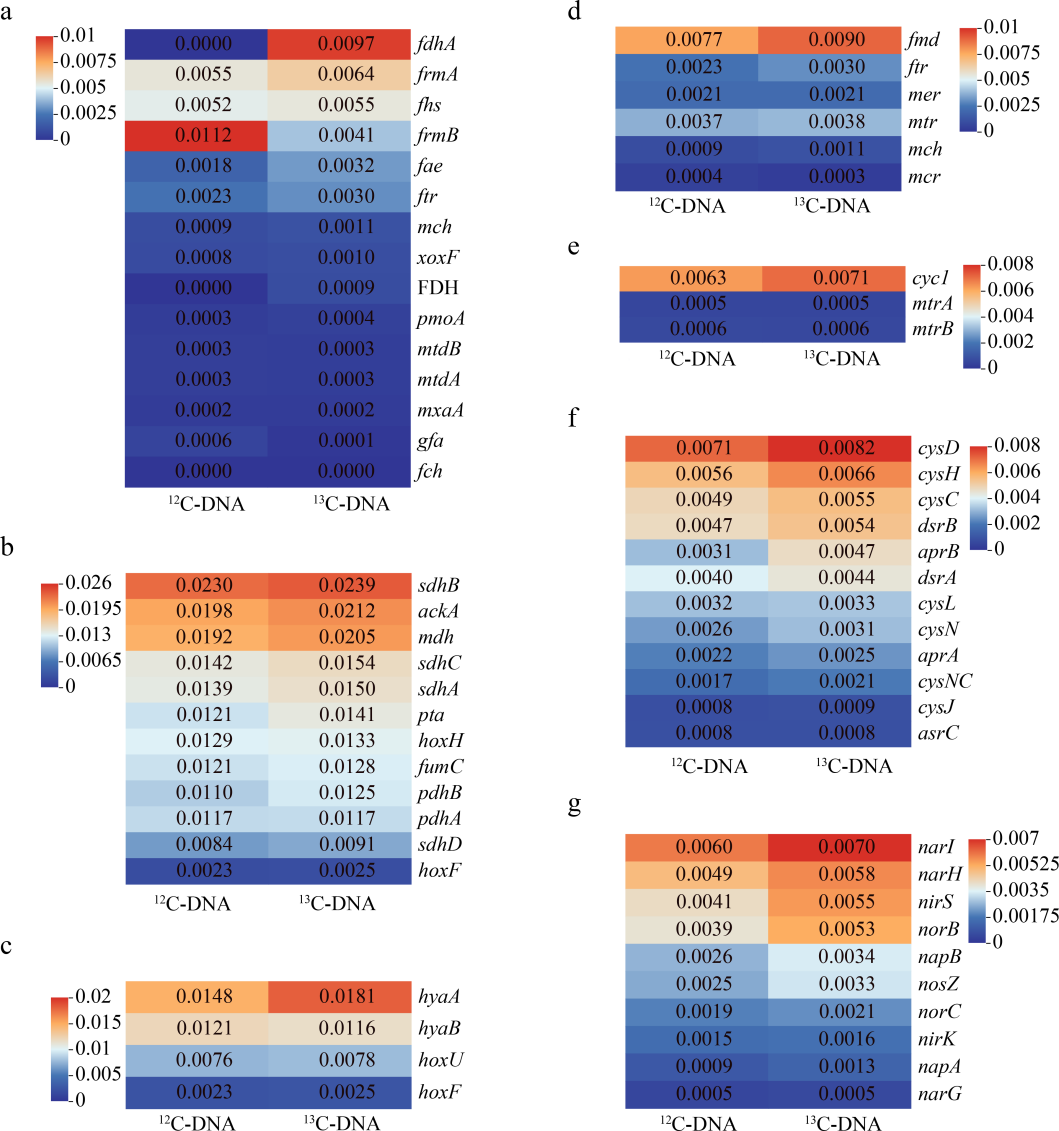
Heatmaps presenting a comparative analysis of the main genes involved in aerobic CH_4_ oxidation (**a**), fermentation (**b**), hydrogen (**c**), anaerobic CH_4_ oxidation (**d**), iron (**e**), sulfate (**f**), and nitrogen (**g**) metabolism in ^12^C-DNA and ^13^C-DNA samples. Gene abundances are expressed as TPM, calculated by normalizing read counts to gene length and sequencing depth, enabling accurate comparison of gene profiles between ^12^C-DNA and ^13^C-DNA samples.

To determine the relative abundance changes in functional genes involved in CH_4_ oxidation and other metabolic processes involving nitrogen, sulfur, hydrogen, iron, and fermentation in ^12^C-DNA and ^13^C-DNA, metagenomic sequencing analysis was performed (Fig. 7). Due to the low DNA yield from SIP fractions, the ^13^C- and ^12^C-labeled DNA fractions from all CH_4_ treatments (^12/13^CH₄ alone and with added electron acceptors) were pooled to obtain sufficient material for metagenomic sequencing. The initial oxidation of CH_4_ to methanol is mediated by the enzyme MMO. The *pmoA* gene exhibited enrichment in ^13^C-DNA compared with ^12^C-DNA. No other pmo or mmoX genes were detected in either sample. The *mxaA* and *xoxF* genes, encoding methanol dehydrogenase (MDH), responsible for the oxidation of methanol to formaldehyde, also showed enrichment in ^13^C-DNA. The *fdhA* gene encoding particulate cytochrome-linked formaldehyde dehydrogenase was highly abundant in ^13^C-DNA, but absent in the ^12^C-DNA. Genes involved in the tetrahydromethanopterin (H_4_MPT)-dependent oxidation of formaldehyde to formate (*fae*, *mtdA*, *mtdB*, *mch*, and *ftr*) and inglutathione-dependent formaldehyde oxidation (*gfa*, *frmA*, and *frmB*) were detected in both ^13^C-DNA and ^12^C-DNA samples. Only the relative abundance of *frmA*, *fae*, *ftr*, and *mch* significantly increased in ^13^C-DNA. The gene encoding formate dehydrogenase (FDH) was only present in ^13^C-DNA samples. Metabolic genes involved in AOM, including *mtr*, *mch*, *ftr*, and *fmd*, were significantly enriched in ^13^C-DNA compared with ^12^C-DNA.

Regarding nitrogen metabolism, the nitrate reductase enzymes encoded by the *narGHI* and *napAB* genes, responsible for NO_3_^-^ to NO_2_^-^, exhibited a significant enrichment in ^13^C-DNA, with enrichment factors of 1.17-fold and 1.32-fold, respectively. The enrichment values were calculated by summing the relative abundances of individual genes (*narG*, *narH*, and *napA*, *napB*) in ^13^C-DNA and comparing them with their corresponding values in ^12^C-DNA. All other genes involved in the complete denitrification process from NO_2_^-^ to N_2_, including *nirK*, *nirS*, *norBC*, and nosZ, were enriched in ^13^C-DNA samples. Several genes related to the sulfate cycle, including the *cysD* gene for SO_4_^2-^ reduction, were also enriched in ^13^C-DNA samples.

Homologs of the *cycl* gene, encoding Cyc1 protein (a high-redox-potential member of the cytochrome c4 family in iron oxidation), were notably enriched in ^13^C-DNA, with an enrichment factor of 1.12. Furthermore, the enrichment of *hyaA* and *hyaB* genes encoding group [NiFe] hydrogenase, along with fermentation-related genes such as *sdhABCD*, *phdAB*, and *mdh* in ^13^C-DNA, highlights active hydrogen metabolism and fermentation pathways.

## DISCUSSION

### Influence of electron acceptors on CH_4_ oxidation and emissions in urban lake sediments

The addition of different electron acceptors led to the reduction in CH_4_ concentrations in the headspace of microcosms, possibly due to their combined effects on CH_4_ oxidation and production. The observed inhibition of methanogenesis here may have significantly contributed to the reduction in CH_4_ concentration. In alpine wetlands, the addition of Fe(III), humic acids, or SO_4_^2-^, either promoted or inhibited CH_4_ production and consumption to varying degrees, leading to similar rates of AOM across different treatments (49). The inhibition of CH_4_ production by electron acceptors involves several biochemical pathways, mainly diverting the electron flow away from methanogenesis (50). For example, SO_4_^2-^, NO_3_^-^, and Fe(III) reduction are energetically more favorable than fermentative processes and methanogenesis, which could outcompete methanogens for common substrates like acetate and H_2_ for energy metabolism (51, 52). The increased abundance of denitrifying bacterium *Denitratisoma*, SO_4_^2-^-reducing bacteria, and Fe(III)-reducing bacteria *Geobacter*, along with the increased relative abundance of functional genes related to nitrogen, sulfur, and iron metabolism in total ^13^C-DNA, further supports this deduction (Fig. 3 and 7).

Additionally, electron acceptors may exert certain toxic effects on microbial communities. For example, high concentrations of sulfide, produced during SO_4_^2-^ reduction, can significantly inhibit CH_4_ production by creating toxic conditions for methanogenic archaea (53). NO_3_^-^ addition has been found to substantially reduce CH_4_ production and suppress methanogenic populations in freshwater ecosystems (54), with intermediate NO_2_^-^ that further inhibited methanogenesis (55). The introduction of electron acceptors can also increase the redox potential, inhibiting key enzymes involved in the CH_4_ production pathway, such as methyl-coenzyme M reductase (56).

CH_4_ oxidation rates significantly increased in our experiments with the addition of NO_3_^-^, aligning with previous findings in a temperate eutrophic lake and paddy soil, which demonstrated that NO_3_^-^ addition can effectively stimulate CH_4_ oxidation rate (57, 58). The stimulation was correlated with an increase in *pmoA* gene copy numbers and methanotroph abundances, implying NO_3_^-^-dependent CH_4_ oxidation. In particular, *Denitratisoma*, enriched in the CH _4_+ NO_3_^-^ treatment, was found to play important roles in NO_3_^-^-dependent CH_4_ oxidation (59). Moreover, many strains of enriched *Bradyrhizobium* possess denitrification genes and pathways (60), potentially linking them to both nitrogen and CH_4_ metabolism as proposed previously (61, 62). The stimulated *Methylocystis* species has also been reported to be capable of complete denitrification from NO_3_^-^ to N_2_ under anoxic conditions, using methanol as a growth substrate (63). Moreover, the addition of NO_3_^-^ stimulated the total CO_2_ production (see Fig. S1 at https://doi.org/10.6084/m9.figshare.29396813), indicating its involvement in the decomposition of organic matter, further underscoring the role of NO_3_^-^ in regulating the carbon cycle in urban lake sediments.

Fe(III) and SO_4_^2-^ showed inhibitory effects on ^13^CO_2_ production, suggesting minimal or negative impacts on CH_4_ oxidation under anoxic conditions. It is important to note that the assimilation of some ^13^CH_4_ and its ^13^C-labeled intermediates into microbial biomass may result in underestimating the ^13^CO_2_ production rates (64). For example, certain methanotrophic bacteria like *Methylomicrobium* alcaliphilum strain 20Z can aerobically oxidize CH_4_ coupled with fermentation, excreting CH_4_-derived compounds such as formate and acetate (65). Metagenome-assembled genomes (MAGs) of *Methylococcales* and *Methylobacter* in anoxic lake water have revealed the presence of genes encoding fermentation pathways (66, 67). The enrichment of fermentation-related genes in ^13^C-DNA suggests that active methanotrophs in the sediment may use fermentation-based methanotrophy in anoxic environments, as demonstrated by a recent study in a permanently stratified freshwater lake (37).

The simultaneous detection of ^13^CO_2_ production alongside Fe(III) and SO_4_^2-^ reduction confirmed CH_4_ oxidation under Fe(III) and SO_4_^2-^-reducing conditions. The calculated molar electron ratio of Fe(III) reduced to Fe(II) relative to ^13^CO_2_ production in sediments far exceeds the stoichiometric ratio expected from AOM coupled with iron reduction, indicating that CH_4_ is not the sole electron donor for iron reduction. Additionally, the added Fe(III) may influence CH_4_ oxidation through other pathways. Fe(III) minerals not only can act as electron acceptors but also influence various biochemical processes in the environment through chemical adsorption and catalysis (68). Fe concentrations can cause niche differentiation and activity of methanotrophic bacteria, potentially affecting CH_4_ oxidation (69, 70). Fe(III)-dependent AOM has been identified in iron-rich lake sediments and As-contaminated groundwater aquifers (16, 31, 71). The sediment sampling site, located in the Sichuan Basin, is characterized by its unique purple soil with a naturally high iron content (*in situ* Fe(III) concentration: 20.1 mM), likely naturally enriched with Fe-cycling microorganisms, such as the presence of *Geobacter* and *Deferrisoma* (Fig. 3h). These bacteria may act as partners of CH_4_-oxidizers facilitating key processes involved in the CH_4_ oxidation under iron-rich conditions (72).

Furthermore, although the detection of ^13^CO_2_ indicates that CH_4_ oxidation did occur in anoxic microcosms, the relatively small amount of ^13^CO_2_ compared with the total CH_4_ decrease suggests that CH_4_ oxidation alone cannot account for the observed CH_4_ dynamics. Future studies incorporating multiple-isotope substrate labeling and multivariable analyses are needed to distinguish the relative contributions of electron acceptor-driven methanogenesis inhibition and CH_4_ oxidation stimulation.

### Both aerobic and anaerobic methanotrophs are involved in CH_4_ oxidation in lake sediments under anoxic conditions

Our study demonstrates diverse microbial involvement in CH_4_ oxidation in the anoxic lake sediments. The most abundant active CH_4_-oxidizer MBG-D*,* an uncultured archaeal lineage within the *Thermoplasmatales* order, is often found in a variety of freshwater and marine habitats (73–75), especially in the AOM zone (74, 76). Genomic and metatranscriptomic analyses suggest that MBG-D may exhibit mixotrophic metabolisms, involving CH_4_ oxidation due to their metabolic versatility and associations with other archaeal groups in methane-rich environments (77). The identified key taxon MBG-D further supports its potential importance in CH_4_ oxidation in the sediments. This further highlights the ecological relevance of MBG-D in CH_4_ cycling and underscores the need for future enrichment and genomic characterization of this archaeal lineage.

*Methylocystis*, which assimilates ^13^CH_4_-derived carbon, is known to thrive in oxygen-deficient environments, such as in floodplain sediments (78), denitrifying bioreactors (79), and paddy soil (80). Some species of *Methylocystis* are classified as facultative methanotrophs, capable of using different carbon substrates, such as methanol, ethanol, and acetate (79, 81). The fermentative metabolism of these *Methylocystis* sp. might serve as an oxygen-saving strategy to support their microbial metabolism (65). Certain *Methylocystis* strains, such as *Methylocystis* sp. strain SC2, are capable of complete denitrification from NO_3_^-^ to N_2_, offering a mechanism to overcome oxygen limitation (63). Moreover, nitrogen fixation genes have been detected in the genome of some *Methylocystis* sp. (78), potentially enabling nitrogen fixation and supporting microbial growth. This may confer an advantage for *Methylocystis* under our nitrogen-limited condition, as the inorganic nitrogen was undetectable by the end of the anoxic incubations (see Fig. S4 at https://doi.org/10.6084/m9.figshare.29396813). A recent study also indicated that *Methylocystis* likely uses riboflavin and *c*-type cytochromes as electron carriers for ferrihydrite reduction (80), which may explain the identification of the labeled *Methylocystis* in the ^13^CH_4_ + Fe(III) treatment.

Another active CH_4_ consumer during the anoxic incubation is *Methylobacter*, commonly found in lakes, wetlands, and rice paddies (82–85). The aerobic methanotrophic *Methylobacter*, especially facultative species, have been shown to actively oxidize CH_4_ in the anoxic waters of Lacamas Lake and Northwestern Siberian Lake, often in association with denitrifiers and/or iron-cycling reinforcing partners (67, 86). Previous genomic and cultured studies verified that members of *Methylobacter* sp. harbor genes for mixed-acid fermentation, denitrification, or H_2_ production (67, 87), which have been proposed as strategies for energy conservation in aerobic methanotrophs under O_2_-limited or anoxic conditions (65, 88–90). The adaptations of *Methylobacter* sp. to low O_2_ environments are also linked to their strong O_2_ affinity (<10 µM O_2_) (87). Those might explain their proliferation during the anoxic incubation. This adaptation was further supported by findings in the deep anoxic sediment layers of an iron-rich lake, where *Methylobacter*, as part of the methanotrophic bacteria consortium, exhibited transcriptional activity and was potentially involved in CH_4_ oxidation (24).

The CH_4_ oxidation mechanisms of “*Ca*. Methylomirabilis Sh765B-TzT-35 and Z114MB74” remain poorly understood. The assimilation of “*Ca*. Methylomirabilis’ Sh765B-TzT-35” was previously verified in a SIP study using wet forest soil (91). Additionally, “*Ca*. Methylomirabilis Sh765-TzT-35” has been found in the NO_3_^-^-depleted, anoxic layers of many Central Swiss lakes (92). The NC10 phylum member *“Ca*. Methylomirabilis limnetica” has been reported to likely harbor genes encoding a complete CH₄ oxidation pathway and an incomplete denitrification pathway (93). Moreover, the addition of NO_3_⁻ significantly enriched ‘*Ca*. Methylomirabilis Sh765B-TzT-35’ and ‘Z114MB74’, suggesting aerobic CH_4_ oxidation coupled with denitrification. However, further investigation, for example, of to-be-established enrichment cultures is required to elucidate their specific CH_4_ metabolism pathways.

*Methanobacterium*, a genus belonging to the family *Methanobacteriaceae*, consists of strictly anaerobic archaea (94). The labeling of *Methanobacterium* observed in ^13^CH_4_-amended treatments is likely attributable to cross-feeding on ^13^CO_2_ released from anaerobic CH_4_ oxidation by other methanotrophs. As a hydrogenotrophic methanogen, *Methanobacterium* can assimilate ^13^CO_2_ via CO_2_ reduction with H_2_, a process that has been reported in prior isotope probing studies as a common mechanism of indirect labeling in methane-cycling microorganisms (95, 96).

### NO₃⁻ stimulated a more diverse community of CH_4_-oxidizing microorganisms

The presence of NO,_3_^-^ supported by a diverse community of methanotrophs, NO_3_^-^ can facilitate processes such as NO_3_^-^- or NO_2_^-^-dependent CH_4_ oxidation, which may promote the proliferation of more diverse microbial groups in anoxic sediments (59). For example, *Methyloversatilis* has been found to be involved in methanol- and ethanol-associated denitrification (97). Many *Methyloversatilis* spp., such as *Methyloversatilis* discipulorum, with 100% similarity to the enriched *Methyloversatilis* ASVs, are known to be capable of denitrification (98).

The stimulated *Methylomonas* in this study is phylogenetically close to *Methylomonas lenta* (with 16S rRNA gene similarity of 97.68%). *Methylomonas lenta*, originally isolated from a cow stable slurry pit, has demonstrated tolerance to high concentrations of NO_3_^-^ (up to 100 mM), suggesting its potential to metabolize NO_3_^-^ (99). Incomplete NO_3_^-^ reduction by aerobic methanotrophs, such as *Methylomonas* denitrificans strain FJG1, has been demonstrated under hypoxia conditions (100). *Methylocaldum*-affiliated microorganisms have also been found to tolerate high ammonium concentrations in O_2_-limited environments (101). This characteristic may enhance the activity of these microorganisms in response to NO_3_^-^ addition. Additionally, members of *Methylosoma* and *Methylospira*, labeled in the CH_4_+NO_3_^-^ treatments, are shown to prefer microoxic conditions in wetland or lake sediment (102, 103). These findings validate the deep association between NO_3_^-^ reduction and CH_4_ oxidation in freshwater environments, supported by various active CH_4_ oxidizers in anoxic urban lake sediments.

### Conclusions

This study reveals that adding different electron acceptors influenced CH_4_ oxidation processes and shaped active methanotrophic communities in anoxic urban wetland sediments. We identified key methanotrophs, including both aerobic and anaerobic groups such as MBG-D, “*Ca*. Methylomirabilis,” and *Methylobacter*. Notably, NO_3_^-^ significantly stimulated CH_4_ oxidation, promoting a broader diversity of methanotrophic communities. SO_4_^2^⁻ and Fe(III) mineral amendments favored the growth of *Methylocystis*. These results, combined with metagenomic analysis, further demonstrate the intricate interactions between CH_4_ oxidation and various biogeochemical metabolisms in lake sediments, particularly denitrification. Future research is needed to explore the underlying metabolic pathways and regulatory mechanisms of methanotrophs under varying electron acceptor conditions, as well as their implications for mitigating CH_4_ emissions in urban wetlands.

## Data Availability

The sequences were deposited into the NCBI Sequence Read Archive (SRA) database with the accession number PRJNA1177530.
